# Variability in the Control of Type 2 Diabetes in Primary Care and Its Association with Hospital Admissions for Vascular Events. The APNA Study

**DOI:** 10.3390/jcm10245854

**Published:** 2021-12-14

**Authors:** Sara Guillen-Aguinaga, Luis Forga, Antonio Brugos-Larumbe, Francisco Guillen-Grima, Laura Guillen-Aguinaga, Ines Aguinaga-Ontoso

**Affiliations:** 1 Azpilagaña Health Center, Navarra Health Service, 31008 Pamplona, Navarra, Spain; sguillen.4@alumni.unav.es; 2 Department of Health Sciences, Public University of Navarra (UPNA), 31008 Pamplona, Navarra, Spain; ablm649@gmail.com (A.B.-L.); ines.aguinaga@unavarra.es (I.A.-O.); 3 Department of Endocrinology, Hospital Universitario de Navarra, 31008 Pamplona, Navarra, Spain; lluis.forga.llenas@navarra.es; 4 Instituto de Investigación Sanitaria de Navarra (IdiSNA), 31008 Pamplona, Navarra, Spain; 5 Clínica Universidad de Navarra, 31008 Pamplona, Navarra, Spain; lguillen@unav.es; 6 CIBER-OBN, Instituto de Salud Carlos III, 28029 Madrid, Spain

**Keywords:** healthcare disparities, diabetes mellitus, Type 2, vascular diseases, primary health care, cohort

## Abstract

Type 2 diabetes (T2D) is associated with increased cardiovascular morbidity, mortality, and hospital admissions. This study aimed to analyze how the differences in delivered care (variability of glycosylated hemoglobin (HbA1c) achieved targets) affect hospital admissions for cardiovascular events (CVEs) in T2D patients. Methods: We analyzed the electronic records in primary care health centers at Navarra (Spain) and hospital admission for CVEs. We followed 26,435 patients with T2D from 2012 to 2016. The variables collected were age, sex, health center, general practitioner practice (GPP), and income. The clinical variables were diagnosis of T2D, weight, height, body mass index (BMI), blood pressure (BP), HbA1c, low-density lipoprotein cholesterol (LDL-C), smoking, and antecedents of CVEs. We calculated, in each GPP practice, the proportion of patients with HbA1c ≥ 9. A non-hierarchical K-means cluster analysis classified GPPs into two clusters according to the level of compliance with HbA1C ≥ 9% control indicators. We used logistic and Cox regressions. Results: T2D patients had a higher probability of admission for CVEs when they belonged to a GPP in the worst control cluster of HbA1C ≥ 9% (HR = 1.151; 95% CI, 1.032–1.284).

## 1. Introduction

Type 2 diabetes (T2D) is a highly prevalent disease associated with increased cardiovascular (CV) morbidity, mortality, and hospital admissions [1,2,3]. CV risk factors in patients with T2D are more prevalent than in the general population. CV risk factors can be modifiable and non-modifiable. Modifiable factors include glycemic control, smoking, obesity, hypertension, and dyslipidemia [4,5]. Non-modifiable factors comprise a family history of CV disease, years of T2D evolution, race, gender, age, age at T2D debut, and antecedents of cardiovascular events (CVEs) [6]. In T2D patients, low socioeconomic status is associated with higher mortality [7,8,9,10,11,12,13].

Glycosylated hemoglobin (HbA1c) is a marker used in detecting and monitoring diabetes. HbA1c relates to the risk of vascular complications in patients with and without diabetes [14]. Elevated HbA1c predicts CVEs [15].

Worldwide compliance with the recommendations for treatment of T2D patients is not adequate. Approximately only half of the population have control levels of HbA1c < 7% [16,17,18]. BP control and LDL-C can be used for evaluating T2D follow-up because of their association with cardiovascular events (CVEs) [19,20]. Guidelines recommend maintaining BP < 140/90 mg/dL and LDL-C levels < 100 mg/dL [2,4]. Despite pharmacological advances and new devices for treating and controlling T2D, clinical practice still varies.

There is variability among countries, regions, and physicians who monitor T2D patients Patient care influences risk factors, control, and treatment compliance [21,22,23,24]. The evolution of quality indicators in patients with T2D has been evaluated, including complications such as foot ulcers, amputations, and retinopathy [25,26]. However, little has been studied regarding their influence on the development of CVEs. It is interesting to know the level of compliance achievable. Reaching full compliance with the indicators does not depend solely on the measures proposed by doctors. There are factors that they cannot control and that sometimes rely on their patient’s adherence to treatment.

Primary health care centers (PHCCs) care for T2D patients in Spain. All PHCCs belong to an administrative unit, a health area with an assigned referral hospital. In Navarra, there are three health areas (Pamplona, Tudela, and Estella). PHCCs coordinate with their referral hospital or outpatients’ endocrinology services in complex patients. Interventions in health centers aim to treat and control the disease and act on modifiable factors, following the recommendations of the different scientific societies [2,4,20].

In Navarra, PHCCs use a computerized medical records system, which was implemented in 2004 and has been in general use since 2012. Our study is part of the “APNA Study” (Navarra Primary Care Study). This prospective multipurpose cohort analyzes data from the computerized medical record registry of Navarra. Many studies are conducted with the data from this registry, which endorses the data quality in terms of T2D [27,28,29,30].

This study aimed to analyze how the variability of the control of HbA1c influences hospital admissions for CVEs.

## 2. Materials and Methods

This prospective cohort study was undertaken in Navarra. Navarra is a region in northern Spain with a national health service in which access to healthcare is universal. All inhabitants have free access to PHCCs and hospitals. The development of the APNA study is as follows: In 2004, the clinical records of seven health centers in Navarra were computerized. The clinical records included all of the people assigned to those seven PHCCs. In 2012, the computerized registry of clinical records was expanded to all of the PHCCs of Navarra. The database collects all primary care medical records of the entire population of Navarra. In 2012, the data of paper medical records from 2004 to 2011 of all of the health centers were added to the computer database. We extracted from the database all patients who had a T2D diagnosis recorded.

We analyzed the electronic records with the clinical variables that doctors or nurses recorded in coded form during patient visits. We also collected the analytical results from the laboratories that were automatically included in the clinical record. We extracted the first hospital admission date with a CVE as the main reason for admission during the follow-up period from the hospital discharge registration system. We followed patients until an event occurred or until 31 December 2016.

The CVEs included ischemic heart disease (International Classification of Diseases (ICD) codes: 410–414; ICD10: I20–I25) and cerebrovascular disease (ICD: 430–438; ICD10: I60–I69).

### 2.1. Sample Size

The mean population of the dynamic cohort from 2012 to 2016 was 480,637 (243,129 females and 237,508 males). The total number of T2D patients followed up was 26,435.

### 2.2. Study Variables

In all PHCCs, a population group called “general practitioners practice” (GPP) or “quota” is assigned to a team formed by a family physician and a nurse responsible for their care. We recorded each patient’s GPP. We studied 385 GPPs with a mean population of 1420 people and a standard deviation (SD) of 342, ranging between 508 and 2165 persons.

We collected each year of the cohort, age, sex, health center, GPP, and T2D diagnoses made by physicians. In Spain, an income threshold of 18,000 euros/year was considered an indicator of low socioeconomic status used to establish pharmaceutical co-payment. Low income (less than 18,000 euros) was extracted from the records. The clinical variables collected were weight (kilograms), height (meters), body mass index (BMI) (weight/height^2^), systolic and diastolic BP (mmHg), HbA1c%, LDL-C (mg/dL), and smoking during the follow-up period. Patients with a BMI ≥ 30 were classified as obese. The values of the clinical variables before admission were collected. In the case of non-admission, the first value of the year was collected. The antecedents of previous hospital admissions for CVEs were also recorded. The reference for lipid control was LDL-C < 100 mg/dL [2,4]. We extracted patients’ medications from the database.

### 2.3. Statistical Analysis

For each year, we calculated four control indicators of patients with T2D: Percentage with HbA1c < 7%; percentage with HbA1c ≥ 9%; percentage with BP < 140/90 mmHg; percentage with LDL-C below the target. We used the 2012 Navarra ”Primary Health Care Guidelines for Type 2 Diabetes” currently in force [31]. In patients with T2D without cardiovascular disease, the primary prevention target was LDL-cholesterol < 100 mg/dL. For patients with T2D and history of CVEs, the target was LDL-C < 70 mg/dL (secondary prevention) [31]. We analyzed the mean values of each indicator during the years of follow-up. We show the percentage of compliance and the coefficient of variation to identify the variability of the control indicators between GPPs. In addition, using a non-hierarchical K-means cluster analysis, we classified GPPs into two clusters according to the level of compliance. To calculate the probability of a patient meeting an indicator above the five-year follow-up average, we used non-conditional logistic regression. We calculated odds ratios, with 95% confidence intervals, between patients included in the GPP group with the highest level in each indicator versus the lowest group. We adjusted the models for age, sex, low-income status, and a previous CVE. We computed the Charlson comorbidity index, an index that has been validated for CVEs [32,33,34].

**Figure 1:**
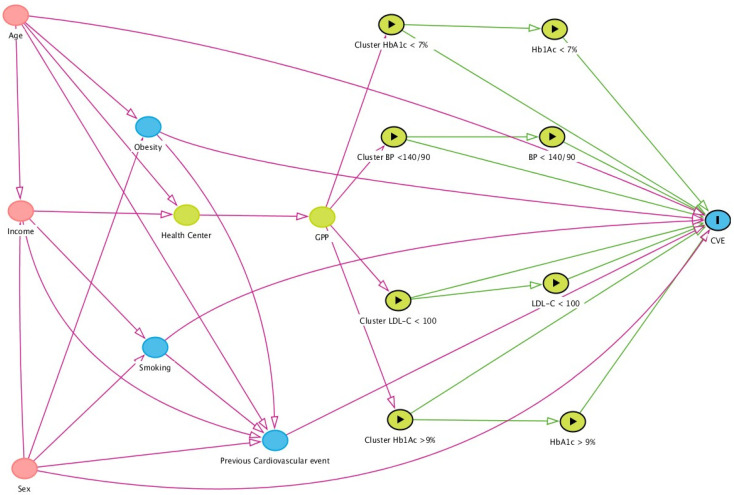
Directed acyclic graph (DAG) of the effect of the cluster of HbAc1 ≥ 9% (exposure) on cardiovascular events (outcome). Ancestors of exposure and outcome 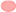, ancestors of outcome 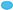, ancestors of exposure 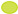, exposure 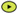, and outcome 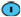. Based on DAGitty version 3.0.

Introducing unnecessary control variables into a multivariate model can lead to bias. DAGs (directed acyclic graphs) are a handy tool to avoid this. DAGs allow drawing a theoretical map of the relationships among variables determining what variables should be controlled in a multivariate model, avoiding bias [35]. In a DAG, arrows represent known causal effects. DAGs allow defining what variables should be handled as cofounders. Using DAGitty statistical software [36], we studied the relationship between the exposure cluster of HbA1c ≥ 9% with CVEs and the potential confounding factors sex, age, low income, obesity, smoking, and CVE antecedents (Figure 1).

The DAG shows that age and income determined where people lived and influenced the health center they were linked to and the GPP they belonged to. GPPs were associated with preventive measures clusters. Age and sex were related to the appearance of CVEs and influenced the antecedents of CVEs, obesity, and current smoking. We observed that the cluster directly affected CVEs and the control measure that affected the outcomes.

**Figure 2:**
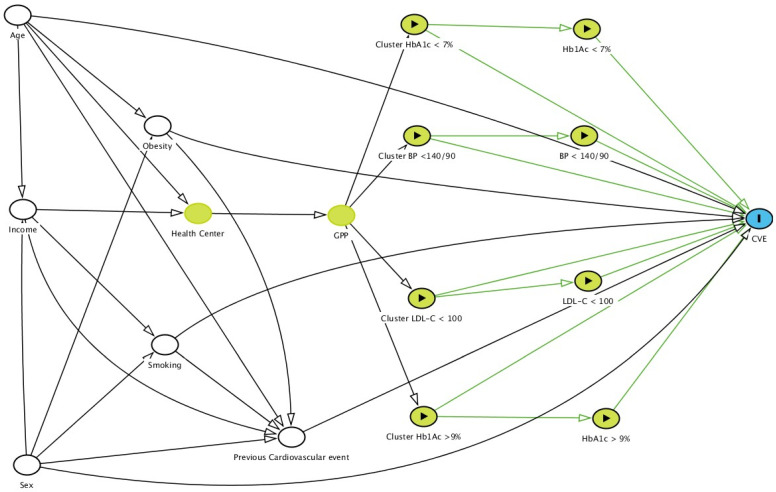
Adjusted directed acyclic graph (DAG) of the effect of the cluster of HbA1c ≥ 9% (exposure) on cardiovascular events (outcome). Adjusted variables 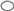, ancestors of exposure 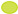, exposure 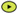, and outcome 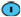. Based on DAGitty version 3.0.

The DAGs also showed the effects of obesity, smoking, and antecedents of CVEs on the incidence of admissions for a CVE (Figure 1 and Figure 2). The DAG software indicated that it was necessary to adjust by sex, age, income, obesity, and current smoking to study the effects of clusters and controls in CVEs (Figure 2).

Finally, we used Cox regression to estimate the excess risk of admissions for a CVE associated with T2D control and belonging to the GPP cluster with the highest score in the control indicators, adjusted for age, sex, low income, obesity, current smoking, history of a CVE before admission, anti-diabetes, lipid-lowering, and antihypertensive medications.

### 2.4. Inclusion and Exclusion Criteria

Inclusion criteria: All patients registered in Navarra with T2D were included.

Exclusion criteria: We excluded patients from other regions who came to the health center because they were ill while traveling. We also excluded non-permanent residents (visitors, students, or seasonal workers). We excluded 17,342 patients who belonged to 96 small GPPs (less than 500 people). These excluded GPPs had a mean size of 317 persons with a standard deviation of 130. These GPPs considered outliers represented less than 3% of the GPPs and were excluded. Of the remaining 480,637 patients in the cohort, we selected all of the patients diagnosed with T2D diabetes for the follow-up.

### 2.5. Study Outcome

The outcome was the first hospital admission for a CVE during the follow-up period.

### 2.6. Ethical Aspects

Patient data were anonymized. The GPPs and the data of the GPP physicians and nurses were anonymized to prevent their identification. We did not have access to physicians’ and nurses’ variables (age, sex, years of experience, years working in the same GPP, training, or teaching activity).

The Spanish Agency for Medicines and Health Products of the Ministry of Health, Social Services, and Equality authorized the study under code ABL-MET-2013-01 on 9 December 2013. The Clinical Research Ethics Committee of Navarra (CEIC) approved the study with the number 3/2014 on 26 March 2014.

## 3. Results

### 3.1. Descriptive Statistics

Table S1 shows the descriptive indicators of the population. In 2012, the prevalence of T2D was 4.14%. There were 2258 CVEs, and of those, 1535 (67.98%) were ischaemic cardiopathies and 723 (32.02%) were strokes.

### 3.2. Multivariate Cluster Analysis of GPPs

A GPP was in the worst control group of HbA1C ≥ 9% when more than 15.87% of T2D patients in the GPP had HbA1c ≥ 9%. The range in the worst control group was from 15.87% to 50%. Poor HbA1c control ≥ 9% was likely in patients assigned to a GPP in the worst control group of HbA1c ≥ 9% (OR: 1.729; 95% CI, 1.631–1.834) (Table S2). The mean proportion of patients with HbA1c ≥ 9% was 21.42% in the GPPs of the worst group versus 10.12% in the best (Table S3). A GPP was in the best group of HbA1c < 7% when more than 38.24% of patients in the GPP had HbA1c < 7%. The range in the best control group was from 38.24% to 100%. The differences between the GPPs with poor glycemic control cannot be fully explained by the proportion of people with a low income. In the GPPs of poor glycemic control (Hb1Ac ≥ 9%) the proportion of people with low income was higher (4.44%) than in the GPPs of better control (3.61%) (chi-square = 249,056, df = 1, *p* < 0.001). Although highly significant, this difference is of a small magnitude (0.80%).

### 3.3. Cox Regression

Table 1 presents the Cox model of hospital admissions for CVEs. There were two independent risks. One was associated with the individuals (having HbA1c ≥ 9%) and the other with the GPPs (being a patient of a GPP with more than 15.87% of patients with HbA1c ≥ 9%). Patients with HbA1c ≥ 9% had an HR of 1.339 (95% CI, 1.070–1.676). T2D patients who belong to GPPs in the worst control group of HbA1c had a HR of 1.119 (95% CI, 1.003–1.250) of having a CVE. Having HbA1c < 7%, blood pressure < 140/90 mmHg, and LDL-C below the primary or secondary prevention thresholds were protective factors, with HRs of 0.736 (95% CI, 0.632–0.856), 0.732 (95% CI, 0.628–0.853), and 0.240 (95% CI, 0.196–0.294).

In the subgroup of subjects with poor glycemic control (HbA1c ≥ 9), we did not detect any association between BP and LDL-C with hospital admission for CVEs (Table S5).

## 4. Discussion

### 4.1. Strengths and Limitations

Our study has several strengths. First, we studied the entire population of Navarra, an area in the north of Spain with over 600,000 inhabitants. This minimized the risk of selection bias.

We used cluster analysis to classify GPPs’ compliance with the indicators. Cluster analysis creates groups to minimize intragroup variability and maximize extra group variability. This analysis allowed us to correct the drawbacks of more straightforward methods, such as deciles or quartiles and others that include individuals with outliers that may produce bias.

Our population was so large that slight differences, without clinical relevance, could be significant. Therefore, using the hazard ratio or odds ratio to measure the association’s strength allowed us to visualize better the most clinically relevant differences between the different GPPs in terms of compliance with the quality indicators.

We did not know how many years general practitioners were in a GPP. In the future, it would be interesting to analyze in other studies the continuity of the same physician in each GPP. Another limitation was that the database did not include deaths by CV diseases.

Our study was carried out based on the electronic records of health professionals during patient care, being a faithful reflection of clinical practice. This is one strength of this type of study. However, it could have biases because of the limitations in the quality of the records. Studies with large administrative databases often show high accuracy in the diagnostic and treatment aspects. Still, they have underreported or lack information on crucial aspects of the diet or physical activity. This lack of information on lifestyle produces misclassification problems that may affect the outcome of interest. In contrast, in many traditional “ad hoc” epidemiological studies, there is a great deal of information on risk factors. However, the clinical data are incomplete or of lower quality.

Administrative databases may not obtain reliable estimates of the effects of preventive actions because of unmeasured confounding factors [37]. This can occur in both longitudinal and cross-sectional studies [38]. Our study’s lifestyle or clinical examination data may have underreported smoking, BP, and weight measurements. However, our results were similar to those of previous studies in Spain [18,39,40,41,42,43,44,45,46,47]. Laboratory data were collected automatically, accurately reflecting what had been requested for each patient. The registry of Navarra was rated as the highest quality among the 17 regional health services in Spain in patients with T2D [42]. In Navarra, studies have shown the usefulness of electronic records for assessing the quality of care of patients with T2D [7,27,29]. This study could only access data on hospital admissions for CVEs, but not mortality data. Future studies would be of great interest in analyzing the risk of CV mortality in patients with T2D [39].

It would have been interesting to include the duration of diabetes in the analysis [47]. However, this was not possible. In the year 2012, primary care medical records were computerized in Navarra. The computer system assigned all patients registered for the first time in the system (prevalent or incident cases) the date of the first consultation as the date of diagnosis of T2D. This occurred in 2012 for 20,732 patients with T2D of the 26,389 in the cohort. Therefore, this variable could not be used in the analysis.

### 4.2. Variability in Control Indicators

HbA1c is a marker of the average blood glucose concentration that is linearly related to the risk of vascular complications. HbA1c is very useful for the diagnosis of diabetes. Its value is increasingly discussed as a treatment objective, because the average glucose and blood glucose range is becoming critical [48]. Although the HbA1c level is continuously associated with CV risk, different guidelines have recommended HbA1c < 7% as the cut-off point for establishing recommendations for managing T2D [1,4]. Analysis of electronic HbA1c records helps to predict increased emergency department visits and hospital admissions [49]. In Navarra, a cross-sectional study showed that women were less likely than men to achieve HbA1c control targets (59% vs. 61%). Patients under 65 years of age had worse control than the older age groups [27]. Data from our study agree with these results [45,47]. Intra- and inter-GPP variability in the treatment of diabetes could not be explained by differences in the guidelines for the treatment of diabetes. The guidelines were the same throughout Navarra [31]. All GPPs should follow them, but the degree of adherence to these guidelines could change.

The main finding of our study was that patients associated with a GPP in the worst control cluster of HbA1c ≥ 9% had a higher likelihood of having a HbA1c level ≥ 9% by 73%. This is a cause for concern because of the inequity involved.

### 4.3. Variability: The Risk of Admission for a CVE

The most frequent complication leading to hospitalization of T2D was stroke (34.7%), followed by ischemic heart disease (28.7%). Our study provides information on the risk of admission for stroke and ischemic heart disease patients with T2D. However, it has the limitation that it does not include deaths due to these causes. Still, it allows adjusting the risk of admission by age, sex, and low income and the variability due to the physician providing the care. Our study found that the risk of hospital admissions for CVEs increased by 3.4% with each year of age. Other studies have reported a similar annual risk of fatal and nonfatal CVEs between 2% and 5% [3,37,48,49,50,51]. The risk of admission increased if HbA1c had elevated values of ≥9% (HR: 1.665). Our results coincided with other studies indicating an increased CVE risk with elevated HbA1c levels [52,53,54].

We found differences between GPPs with a higher proportion of patients with poor control of HbA1c ≥ 9%. A GPP in a poorly controlled cluster entailed an additional risk of admission for a CVE of 14.7%. This risk is added to the patient’s risk when his HbA1c is uncontrolled (HbA1c ≥ 9%).

### 4.4. Implications for Research and Practice

Variability in clinical practice influences the health of the population. Healthcare workers’ knowledge and interest in specific pathologies affect the control of different health indicators [21,22,24].

Continuity of care is also crucial. Having a primary care physician that knows their patients for many years is a protective factor in admissions and mortality [55,56]. We did not evaluate this item because it did not appear in the database analyzed.

The intra- and inter-variabilities could not be explained by major differences in the guidelines for diabetes treatment in the GPPs. In Navarra, a management unit in the Primary Health Care Directorate (PHCD) established a single clinical practice guideline for the entire region. This guide was based on the ADA (American Diabetes Association) guidelines [4]. In 2012, the last version of Navarra’s guide was elaborated [31]. PHCD implemented training actions to promote adherence and reduce variability in its application. A pharmacy unit from the PHCD also made recommendations consistent with the guide, establishing a follow-up and evaluation of pharmaceutical prescriptions.

With a few indicators, our study provided information on the technical quality achieved in clinical practice in the care of patients with T2D, identifying variability among primary healthcare providers. Cluster analysis allowed us to form homogeneous groups of GPPs, detecting those who achieved better or worse results in routine clinical practice. We became aware of a significant equity problem between healthcare providers.

For confidentiality reasons, we did not have access to physician variables (age, sex, years of experience, years working in the same GPP, training, or teaching activity). This is a limitation of our study and should be the subject of further research to determine the characteristics of the physicians with the best and worst results. However, we did have access to the geographic area and the number of people linked to each GPP. These factors did not influence the appearance of CVEs.

It would have been interesting to know the contribution of diet and physical exercise in cardiovascular events. Unfortunately, this information is not collected in primary care medical records. If this information were gathered and coded by primary healthcare nurses in the future, it would be of great clinical and research interest.

From the GPP location, it is impossible to estimate diet or physical activity characteristics. There is no information on diet and physical activity in the region or health areas. The geographic location of a GPP does not provide helpful information. A PHCC may have up to 12 GPPs and include 10,000 people. Socioeconomic status can influence the geographic area in which an individual lives, and therefore the health center to which they are linked (Figure 1). However, the assignation of individuals to GPPs within a health center is random. Therefore, no information on diet and physical activity can be estimated.

Our main objective was not to identify the causes of the variability but rather the effects on diabetic macroangiopathy. The methods used in this study could help establish incentives through benchmarking strategies that introduce a comparative evaluation of best and most efficient practices. This type of strategy is a method of continuous improvement in various business sectors, including the health sector [57]. The summary is that being a patient of a GPP in the worst group increases the risk of CVEs. Clear policy actions follow from this study. It is necessary to force all GPPs to achieve the same high proportion of HB1Ac control in their patients. It is essential to intervene now to reduce this variability, and it is a question of equity and fairness.

## 5. Conclusions

A significant variability in the control of HbA1c inT2D patients was detected. There was also an important variability in the proportion of patients with good blood pressure and LDL-cholesterol among GPPs. The GPP assigned to a patient had an independent effect on hospital admissions for CVEs. Differences between GPPs are distressing because they affect equity in the healthcare system. It is necessary to determine which factors determine the GPPs with the worst proportion of Hb1Ac control. Benchmarking should be carried out so that all GPPs reach the same level in control indicators.

## Figures and Tables

**Table 1:** Cox regression model to analyze the risk of admission for a CVE in patients with type 2 diabetes according to clusters of GPP HbA1c > 9% and individual levels of HbA1c.

Variable		HRa *	95% CI	*p*
HbA1c ≥ 9% **		1.339	1.070–1.676	0.010
HbA1c < 7% **		0.736	0.632–0.856	<0.001
BP < 140/90 mmHg **		0.732	0.628–0.853	<0.001
LDL-C < 100 mg/dL † or <70 mg/dL ‡		0.240	0.196–0.294	<0.001
Cluster inadequate control HbA1c ≥ 9% **		1.119	1.003–1.250	0.045

* Adjusted hazard ratio (full model in Table S4). ** Average five-year compliance. † Primary prevention. ‡ Secondary prevention.

## Data Availability

The datasets generated for this study are unavailable due to the data protection law.

## Supplementary Materials

The following is available online at https://www.mdpi.com/article/10.3390/jcm10245854/s1, Table S1. Descriptive indicators of the population of Navarra, cohort 2012–2016 (≥18 years) stratified by sex. Table S2. Odds ratios of having met the indicator above the five-year mean. Table S3. Variability of the control indicators by GPPs. Table S4. Final Cox regression model risk of admission for a CVE in patients with type 2 diabetes, adjusted by age, sex, income, history of previous cardiovascular anti-diabetes, lipid-lowering, antihypertensive medications, Charlson index, GGP size, and health area. Table S5. Cox regression: Admission for a CVE in the subgroup of patients with poor glycemic control (HbA1c ≥ 9).
