# Supplementary material for: Variability in the Control of Type 2 Diabetes in Primary Care and Its Association with Hospital Admissions for Vascular Events. The APNA Study
Source: J Clin Med. 2021 Dec 14;10(24):5854. doi: 10.3390/jcm10245854 (PMC8703537; doi:10.3390/jcm10245854)
Supplement: Supplementary file 1 [file jcm-10-05854-s001.zip › jcm-1488385-supplementary.pdf]

Suplementary Materials

Table S1: Descriptive indicators of the population of Navarra, cohort 2012 to 2016 (>=18 years) stratified by sex.

		2012			2013			2014			2015			2016		
		**Total**	**Females**	**Males**	**Total**	**Females**	**Males**	**Total**	**Females**	**Males**	**Total**	**Females**	**Males**	**Total**	**Females**	**Males**
**Population**		473448	237856	235592	478917	240522	238395	483777	245394	238383	483825	245837	237988	483217	246037	237180
**T2D Prevalence, n (%)**		19603 (4,14)	8581 (3,61)	11022 (4,68)	20810 (4,35)	9081 (3,78)	11727 (4,92)	22763 (4,71)	9901 (4,03)	12862 (5,40)	24183 (5,00)	10506 (4,27)	13377 (5,75)	24394 (5,05)	10757 (4,30)	13817 (5,83)
**18-34 Years**		214 (0,17)	104 (0,17)	110 (0,17)	212 (0,18)	104 (0,18)	108 (0,18)	212 (0,18)	104 (0,18)	108 (0,18)	207 (0,18)	101 (0,18)	106 (0,19)	199 (0,18)	107 (0,20)	92 (0,17)
**35 -44 Years**		908 (0,90)	329 (1,10)	579 (0,90)	871 (0,85)	315 (0,65)	556 (1,05)	892 (0,87)	334 (0,67)	108 (1,06)	909 (0,90)	341 (0,69)	568 (1,09)	884 (0,89)	339 (0,70)	545 (1,07)
**45 -54 Years**		2576 (2,99)	832 (3,94)	1744 (2,99)	2642 (3,00)	866 (2,03)	1776 (3,92)	2730 (3,03)	901 (2,04)	1829 (3,98)	2853 (3,11)	950 (2,11)	1903 (4,08)	2833 (3,05)	956 (2,10)	545 (3,98)
**55 -64 Years**		4753 (7,38)	1652 (9,50)	3101 (7,38)	4884 (7,37)	1677 (5,14)	3207 (9,54)	5005 (7,30)	1699 (4,99)	3306 (9,59)	5034 (7,21)	1712 (4,92)	3322 (9,48)	5159 (7,21)	1763 (4,94)	1877 (9,47)
**65 -74 Years**		5455 (11,77)	2344 (13,89)	3111 (11,77)	5778 (11,89)	2403 (9,64)	3375 (14,27)	6491 (12,24)	2668 (9,69)	3823 (14,99)	6939 (12,58)	2785 (9,74)	4154 (15,64)	6937 (12,41)	2765 (9,55)	4172 (15,49)
**75 -84 Years**		4628 (12,64)	2544 (13,46)	2084 (12,64)	5006 (13,31)	2713 (12,64)	2293 (14,21)	5561 (14,91)	2901 (13,48)	2660 (16,86)	5822 (15,91)	2993 (14,19)	2829 (18,24)	5837 (15,96)	2972 (14,12)	2865 (14,45)
**85 and over**		1069 (7,44)	776 (6,55)	293 (7,44)	1471 (8,32)	1003 (8,69)	414 (7,55)	1872 (11,83)	1294 (12,00)	578 (11,48)	2419 (14,71)	1624 (14,54)	795 (15,07)	2545 (14,86)	1675 (14,46)	870 (15,67)
**Age Mean (SD)**	**No T2D**	47,87 (17,94)	49,03 (18,67)	46,70 (17,09)	48,47 (18,13)	49,63 (18,85)	47,30 (17,29)	48,60 (17,92)	49,71 (18,57)	47,44 (17,13)	48,94 (13,35)	47,44 (17,13)	47,79 (17,90)	49,76 (18,74)	50,86 (19,47)	48,62 (17,88)
	**T2D**	66,52 (12,74)	69,19 (13,07)	64,45 (12,07)	67,20 (12,87)	69,89 (13,26)	65,13 (12,16)	67,94 (12,97)	70,58 (13,35)	65,92 (12,29)	68,57 (13,15)	71,24 (13,56)	66,53 (12,46)	68,72 (13,14)	71,27 (13,63)	66,76 (12,40)
**Low Income, (%)**	**No T2D**	3,68	4,22	3,13	3,67	4,21	3,13	3,83	4,36	3,28	3,99	4,55	3,41	4,01	4,58	3,42
	**T2D**	4,57	5,56	3,79	4,72	5,85	3,85	4,83	6,12	3,84	5,09	6,36	4,12	5,26	6,47	4,34
**% Obesity (BMI>=30)**	**No T2D**	25,15	23,82	26,65	24,95	23,65	26,43	24,41	23,06	25,96	24,20	22,86	25,74	23,96	22,63	25,51
	**T2D**	46,04	48,86	43,84	46,50	49,26	44,36	46,85	49,47	44,82	47,14	49,72	45,14	48,08	50,50	46,21
**Current smoker (%)**	**No T2D**	24,75	20,78	29,11	24,36	20,60	28,47	24,09	20,62	27,92	23,75	20,44	27,43	23,57	20,39	27,10
	**T2D**	17,01	8,76	23,50	17,04	9,17	23,16	16,79	9,46	22,48	16,40	9,25	21,94	16,85	9,79	22,29

Table S2. Odds Ratios of having met the indicator above the 5-year mean

Variables	**Sex (male)** OR (95% CI)	**Age** OR (95% CI)	**Low Income** OR (95% CI)	**Hospital admission for CVE** OR (95% CI)	**General practices cluster***** OR (95% CI)
Standardized prevalence of T2D (2016)	1.656* (1.614-1.700)	1.056* (1.055-1.057)	2.111* (1.992-2.237)	1.276* (1.211-1.344)	*1.402** (1.363-1.411)
HbA1c <7%**	1.099 (1.053-1.148)	1.001 (0.999-1.002)	0.801 (0.721-0.890)	0.529 (0.490-0.571)	2.457 (2.291-3.636)
HbA1c ≥9%**	0.951 (0.896-1.009)	0.980 (0.978-0.982)	1.447 (1.279-1.637)	1.354 (1.227-1.495)	1.729 (1.631-1.834)
BP<140/90 mmHg **	0.909 (0.871-0.949)	0.979 (0.978-0.981)	0.865 (0.780-0.961)	0.920 (0.855-0.990)	1.833 (1.757-1.913)
LDL-C <100 mg/dl† or <70 mg/dl ‡	1.186 (1.126-1.250)	1.003 (1.001-1.005)	1.027 (0.915-1.154)	0.300 (0.277-0.324)	1.314 (1.244-1.387)

* Prevalence OR **Average value of % compliance by GPP over the five years ***Adjusted for sex, age, low income † Objective primary prevention LDL-C <100 mg/dl ‡ Objective secondary prevention LDLC < 70 mg/dl

Table S3. Variability of control indicators by GPPs

**Variable**	**Mean**	**Number of highest and lowest scoring clusters**	**Mean (range) in clusters with the lowest score**	**Mean (range) in clusters with the highest score**	**Coefficient Variation between general practices**
Standardized prevalence (2016)	5.74%**	101/327	4.83%†	8.20%†	42.32%
HbA1c < 7% *	47.34%	360/72	23.34% (0%-38.10%)	52.14% (38.24%-100%)%	31.72%
HbA1c ≥ 9%*	15.65%	210/222	10.12% (0%-15.78%)	21.42% (15.87%-50%)-	47.41%
BP < 140/90 mmHg *	52.52%	243/188	41.39% (0%-50.98%)	61.18% (51.11%-100%)	25.45%
LDL-C <100 mg/dl† or <70 mg/dl ‡	46.16%	180/252	38.15% (0%-47.73%)	57.36% (47.83-100%)	28.12%

* Average value of % compliance by general practices over the five years. **Age-standardized prevalence (4 General practices were eliminated due to high outlier prevalence values).† Objective primary prevention LDL-C <100 mg/dl ‡ Objective secondary prevention LDLC < 70 mg/dl

Table S4. Final Cox regression model risk of admission for a CVE in patients with type 2 Diabetes adjusting by Age, sex, income, History of previous cardiovascular anti-diabetes, lipid-lowering, antihypertensive medications, Charlson Index, GGP size, and Health Area.

Variable	HR	95% CI	p
Age	1.014	1.007-1.021	<0.001
Sex (male)	1.289	1.144-1.454	<0.001
Low Income	1.617	1.227-2.131	0.001
History of cardiovascular event prior to admission	9.811	8.441-11.410	<0.001
HbA1c < 7%*	0.736	0.632-0.856	<0.001
HbA1c ≥9%*	1.339	1.070-1.676	0.010
BP <140/90 mmHg*	0.732	0.628-0.853	<0.001
LDL-C <100 mg/dl** or <70 mg/dl ***	0.240	0.196-0.294	<0.001
Cluster indicator HbA1c < 7%	0.991	0.842-1.167	0.916
Cluster indicator HbA1c ≥9% (More than 15.87% of the T2D patients in the GPP with HbA1c ≥9%)	1.119	1.003-1.250	0.045
Cluster indicator BP <140/90 mmHg	1.095	0.981-1.225	0.116
Cluster indicator LDL-C <100 mg/dl** or <70 mg/dl ***	0.958	0.856-1.074	0.463
Current smoker	1.141	0.983-1.324	0.084
Obesity (BMI ≥30)	1.057	0.946-1.181	0.327
Charlson Index	1.168	1.137-1.200	<0.001
Insulin	1.453	1.278-1.651	<0.001
Statins	1.705	1.485-1.957	<0.001
Antihypertensive medications	0.981	0.862-1.115	0.764
GPP size (thousand persons)	1.020	0.837-1.243	0.845
Health Area Pamplona (ref)	1		
Estella	1.098	0.913-1.320	0.321
Tudela	1.055	0.915-1.216	0.462

* Average 5-year compliance. ** Primary Prevention. ***Secondary Prevention

Table S5 Cox regression model to analyze the risk of admission for a CVE in the subgroup of patients with poor glycemic control (HbA1c≥9)

Variable	HR	95% CI	p
Age	1.374	1.220-1.548	0.039
Sex (male)	1.036	1.030-1.041	0.308
Diastolic BP	0.962	0.907-1.021	0.201
Systolic BP	1.004	0.964-1.046	0.857
LDL-C	1.007	0.990-1.025	0.427
